# Thoracic Injury Rule out Criteria in Prediction of Traumatic Intra-thoracic Injuries; a Validation Study

**Published:** 2017-01-10

**Authors:** Setareh Asgarzadeh, Bahareh Feizi, Farhad Sarabandi, Morteza Asgarzadeh

**Affiliations:** 1 Clinical Research Development Center, Amir-Almomenin Hospital, Islamic Azad University, Tehran Medical Sciences Branch, Tehran, Iran.; 2 Emergency Department, Pasteur Hospital, Bam University of Medical Sciences, Bam, Iran.; 3 Harvard T.H. Chan School of Public Health, 667 Huntington Avenue, Boston, MA, 02115.

**Keywords:** Thoracic injuries. decision support techniques, mass chest x-ray, diagnosis

## Abstract

**Introduction:**

Doing Chest X Ray (CXR) for all trauma patients is not efficient and cost effective due to its low diagnostic value. The present study was designed aiming to evaluate the diagnostic accuracy of thoracic injury rule out criteria (TIRC) in prediction of traumatic intra-thoracic injuries and need for CXR.

**Method:**

The present study is a prospective cross-sectional study that has been carried out to evaluate the accuracy of TIRC model in screening blunt multiple trauma patients in need of CXR for ruling out intra-thoracic injuries.

**Results:**

1518 patients with the mean age of 33.53 ± 15.42 years were enrolled (80.4% male). The most common mechanisms of trauma were motor car accident (78.8%) and falling (13.6%). Area under the ROC curve, sensitivity, and specificity of model in detection of traumatic thoracic injuries was 0.95 (95% CI: 0.93 – 0.97), 100 (95% CI: 87.0 – 100), and 80.1 (95% CI: 78.0 – 82.1), respectively. Brier score for TIRC was 0.02 and its scaled reliability was 0.0002.

**Conclusion:**

Findings of the present study showed that TIRC has high accuracy in prediction of traumatic intra-thoracic injuries and screening patients in need of CXR.

## Introduction

Traumatic injuries, as one of the causes of morbidity and mortality, inflict a big financial and social burden on health care systems (1). Meanwhile, thoracic injuries are responsible for 20 -50% of trauma-related mortalities (2). Numerous diagnostic tools exist for evaluating these injuries including computed tomography (CT) scan, chest x-ray (CXR), and ultrasonography accompanied by clinical examination. Currently, CXR is considered as the first diagnostic test in traumatic thoracic injuries (3). However, study findings have shown that doing CXR for all patients is not efficient and cost effective due to its low diagnostic value (4, 5). Therefore, researchers are seeking ways to use this tool only for patients with a higher risk of intra-thoracic injuries. In recent years, 2 clinical decision rules, namely Nexus chest in American population and thoracic injury rule out criteria (TIRC) in Iranian population have been introduced for screening patients in need of CXR following blunt trauma. Based on Nexus chest criteria, if any of the factors including age >60 years, rapid deceleration mechanism (falling from a height over 20 feet or being in a car accident with more than 40 mph speed), chest pain, intoxication, altered level of consciousness, distracting pain, and tenderness to chest wall palpation are present, the patient is at high risk regarding presence of injury and CXR is necessary (6). In TIRC model age >60 years, hemodynamic instability, loss of consciousness, crepitation in auscultation, decreased pulmonary sounds, thoracic skin abrasion, and shortness of breath are factors predicting intra-thoracic injuries (7). These 2 models are just starting to be studied and they need to be validated in various populations. Therefore, the present study was designed aiming to evaluate the diagnostic accuracy of TIRC in prediction of traumatic intra-thoracic injuries and need for CXR.

## Methods

***Study design and setting***

The present study is a prospective cross-sectional study that has been carried out to evaluate the accuracy of TIRC in screening patients in need of CXR in multiple trauma patients presented to the emergency department (ED) of Pasteur Hospital, Bam, Iran, during 1 year in 2014-2015. Protocol of the present study was approved by hospital ethic committee. Written informed consent was obtained from all the patients and the researchers adhered to the principles of Helsinki Declaration throughout the study. This project did not cause any disruption in the routine management of patients.

***Participants***

The study participants consisted of all blunt multiple trauma patients over 15 years old who were conscious and had stable hemodynamic. Exclusion criteria included presence of penetrating chest trauma and not giving consent for participation in the study.

***Data gathering***

Sample selection was done using non-randomized convenience sampling. After obtaining informed consent from the patient or their relative, the study checklist was filled. The checklist consisted of demographic data (age, gender, trauma mechanism), history and physical examination findings (distracting pain, loss of consciousness, tachypnea, chest pain, dyspnea, presence of thoracic skin abrasion due to trauma, tenderness in chest, chest deformity, tenderness in upper abdomen, crepitation in chest auscultation, decreased pulmonary sounds, and presence of crepitation), variables needed for TIRC model, and CXR findings. An emergency medicine specialist was responsible for examining, gathering, and recording of data in various days and working shifts.

Immediately after data gathering, CXR was done for patients in 2 standard views of anterior-posterior and lateral, and the pathological findings (hemothorax; pneumothorax; fracture of rib, sternum, scapula, and clavicle; widened mediastinum; and lung contusion) were recorded.

CXRs were interpreted and recorded by an emergency medicine specialist blinded to the clinical findings of the patients as well as the in-charge physician. To evaluate the accuracy of interpretations by the emergency physician, 5% of the CXRs were randomly selected and given to a radiologist for interpretation (Inter-rater agreement between the radiologist and emergency physician was 100%). It should be noted that the radiologist was blind to both the emergency physician’s interpretation and clinical findings. Final diagnosis of thoracic injury was done based on CXR. At times of suspicion to presence of a hidden injury, Chest CT scan was done. 

***TIRC model variables***

Based on this model CXR is necessary for patients with unstable hemodynamics and loss of consciousness. In addition, conscious patients with stable hemodynamics that meet any of the factors including age >60 years, crepitation in auscultation, decrease in pulmonary sounds, thoracic skin abrasion, and shortness of breath, are categorized in the high risk group regarding probability of intra-thoracic traumatic injuries and should undergo CXR.

***Statistical analyses***

To determine sample size, considering the 6.5% prevalence of positive findings in multiple trauma patients’ CXR (8), a 95% confidence interval (CI) (α = 0.05), 90% power (β = 0.1) and maximum error of 1.5% (d = 0.015) in estimating prevalence of injury, minimum sample size was considered 1043. Data were entered to STATA 11.0 software. CXR findings were reported as frequency and percentage, and were divided into 2 groups of normal and abnormal.

In the present study, to assess the validity of the model, a number of methods were used (9, 10) that included calculating the area under the receiver operating characteristic (ROC) curve, sensitivity, specificity, positive and negative predictive value (PPV/NPV), and positive and negative likelihood ratio (PLR/NLR) with 95% confidence interval (CI). 

To evaluate discrimination, calibration curve was drawn for assessing general calibration, and finally in evaluation of overall performance, Brier score was used for assessing predictive accuracy and predictive reliability.

It should be noted that in calibration curve, the perfect calibration is the reference line that has 0 intercept and slope of 1. The closer the slope and intercept of TIRC model are to 1 and 0, respectively, the more perfect the model is for predicting presence or absence of injury in CXR (11).

## Results

Finally, data of 1518 patients with the mean age of 33.53 ± 15.42 years were gathered (80.4% male). Table 1 shows baseline characteristics of studied patients. The most common mechanisms of trauma were motor vehicle collisions (42.1%) and falling down (28.2%). 401 (26.4%) had chest pain, 107 (7.1%) had chest wall tenderness, and 104 (6.8%) had a thoracic skin abrasion. Based on CXR findings, 33 (2.2%) patients had at least 1 traumatic intra-thoracic injury.

***Discrimination***

**Figure 1:**
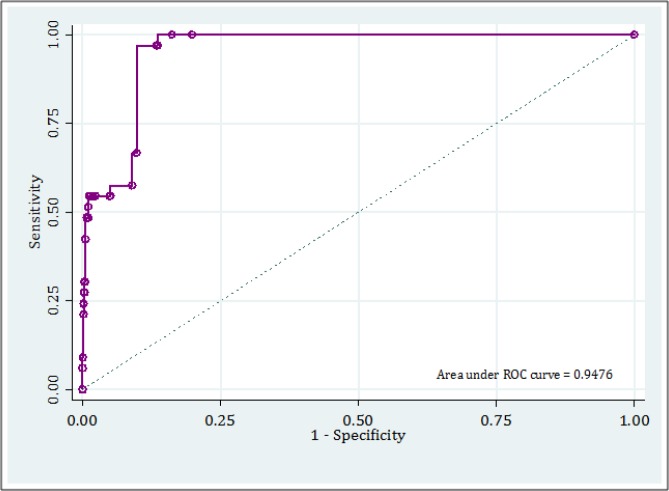
Area under the receiver operating characteristic curve of thoracic injury rule out criteria (TIRC

Area under the curve of TIRC in detection of traumatic thoracic injuries was calculated to be 0.95 (95% CI: 0.93 – 0.97) (figure 1). Considering the presence of at least one of the TRIC risk factors, sensitivity and specificity of model were 100 (95% CI: 87.0 – 100) and 80.1 (95% CI: 78.0 – 82.1), respectively. PPV of the test was 10.1 (95% CI: 7.1 – 14.0) and NPV was 100 (95% CI: 99.6 – 100). PLR and NLR calculated were 5.0 (95% CI: 4.5 – 5.6) and 0 (95% CI: 0.0 – 0.0), respectively (table 2).

**Figure 2:**
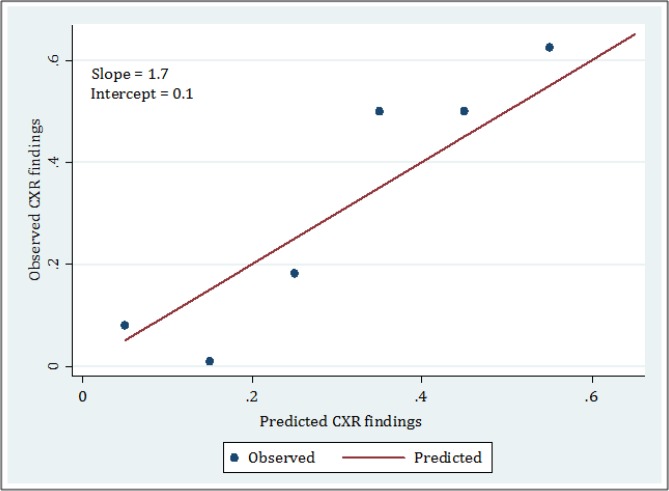
The calibration plot for thoracic injury rule out criteria (TIRC).

Calibration curve of TIRC in detection of an intra-thoracic injury has been presented in figure 2. This scatter plot has an intercept of 0.1 (95% CI: 0.01 -0.19) and a slope of 1.7 (95% CI: 1.3 -1.9) which shows the moderate calibration of this model.

***Overall performance***

Brier score for TIRC was 0.02 and its scaled reliability was 0.0002. These findings are indicative of this model’s high predictive accuracy and reliability.

## Discussion

Findings of the present study showed that TIRC has high accuracy in prediction of traumatic intra-thoracic injuries and screening patients in need of CXR. There was no false negative result in this model and this indicates the proper power of this instrument to rule out intra-thoracic injury following blunt trauma.

**Table 1:** Baseline characteristics of studied patients

**Variable**	**N (%)**
**Age (year)**	
< 60	1468 (96.7)
≥ 60	50 (3.3)
**Gender**	
Male	1220 (80.4)
Female	298 (19.6)
**Mechanism of trauma**	
Motor vehicle collision	1196 (78.8)
Falling down	207 (13.6)
Others	115 (7.6)
**Vital sign (admission time)**	
Systolic blood pressure (mmHg)	119.2±9.1
Diastolic blood pressure (mmHg)	78.2±14.9
SPO2 (%)	97.7±2.9
Respiratory rate (1/minute)	13.6±1.8
**Glasgow coma scale**	
15	1468 (96.7)
Less than 15	50 (3.3)
**Dyspnea**	
Yes	42 (2.8)
No	1476 (97.2)
**Distracting pain**	
Yes	399 (26.3)
No	1119 (73.7)
**Thoracic skin abrasion**	
Yes	104 (6.8)
No	1414 (93.2)
**Chest deformity **	
Yes	8 (0.5)
No	1510 (99.5)
**Chest wall tenderness**	
Yes	107 (7.1)
No	1411 (92.9)
**Crepitation**	
Yes	16 (1.0)
No	1502 (99.0)
**Abdominal tenderness**	
Yes	25 (1.6)
No	1493 (98.4)
**Decrease in pulmonary sounds**	
Yes	37 (2.4)
No	1481 (97.6)
**Chest wall pain**	
Yes	732 (25.20)
No	2173 (74.80)

**Table 2:** Screening performance characteristics of thoracic injury rule out criteria (TIRC) in detection of intra-thoracic injuries

**Characteristics** *	**Value (95% confidence interval)**
**Area under the curve**	0.95 (0.93 - 0.97)
**Sensitivity**	100.0 (87.0 - 100.0)
**Specificity**	80.1 (78.0 - 82.1)
**Positive predictive value**	10.1 (7.1 - 14.0)
**Negative predictive value**	100.0 (99.6 – 100.0)
**Positive likelihood ratio**	5.0 (4.5 – 5.6)
**Negative likelihood ratio**	0.0 (0.0 – 0.0)
**True positive**	33
**True negative**	1190
**False positive**	295
**False negative**	0

* Values given are based on presence of at least one of the following symptoms: age over 60, crepitation, loss of consciousness, decrease in pulmonary sounds, chest wall pain, chest wall tenderness, dyspnea, and skin abrasion.

Based on the findings of this study, if TIRC clinical decision rule was used, only 328 (21.6%) of the 1518 studied patients would undergo CXR. This finding shows that using TIRC will lead to a significant decrease in unnecessary CXRs. In the studied population, 1485 (97.9%) of the CXRs were without any pathologic finding and TIRC predict 1190 (80.1%) of them. This finding is in line with 2 previous studies. In a study by Frouzanfar et al., it was shown that using this tool reduces unnecessary CXRs by 63.5% (7). This rate was 67.7% in Safari et al. study (11). In the Safari et al. study, which was a multi-center one, evaluation of patients was done by different physicians while in the present study all evaluations were done by one emergency medicine specialist. This might be the reason for the higher screening value of TIRC in this study.

In comparing TIRC with Nexus chest model, it is revealed that both models have similar and good value in screening of patients for performing CXR. A study by Rodriguez et al. aiming to validate Nexus chest, indicated the 98.5% sensitivity of this tool in screening traumatic intra-thoracic injuries (12) while this rate was 100% for TIRC. However, it seems that fewer factors in TIRC can be advantageous for using it in clinic. In addition, data such as height of falling and speed of the vehicle at the time of accident (which are required in Nexus chest) are not readily available in many cases, especially in developing countries. However, it is worth noting that validation of nexus chest has only been done in the American population and validation of TIRC has only been done in the Iranian population. Therefore, further studies are needed on both in other settings and geographical areas to ensure their validity.

***Limitations***

One of the limitations of the present study is being carried out in 1 center. Therefore, the results may not be easily generalized. However, since the findings are in line with similar previous studies, It seems that being single centered has not affected the generalizability of the data. Additionally, convenience sampling was used, which raises the probability of selection bias. However, unlike previous studies (11, 12), patient evaluation was done by a single emergency medicine specialist and CXR interpretation was done by another single emergency medicine specialist, which eliminates the effect of difference in assessor in these areas.

## Conclusion:

Findings of the present study showed that TIRC has high accuracy in prediction of traumatic intra-thoracic injuries and screening patients in need of CXR. There was no false negative result in this model and this indicates its proper power to rule out thoracic injury.
